# Allelopathic effects of sesame extracts on seed germination of moso bamboo and identification of potential allelochemicals

**DOI:** 10.1038/s41598-022-10695-x

**Published:** 2022-04-22

**Authors:** Jiancheng Zhao, Zhenya Yang, Jingquan Zou, Qin Li

**Affiliations:** Zhejiang Provincial Key Laboratory of Bamboo Research, Northwest Zhejiang Bamboo Forest Ecological Station, Zhejiang Academy of Forestry, Hangzhou, 310023 China

**Keywords:** Forest ecology, Forestry, Invasive species

## Abstract

The objectives of this study were to investigate the allelopathic effects of sesame extracts of on seed germination of moso bamboo, and to isolate and identify the potential allelochemicals. A factorial design with three organs (root, stem and leaf) and five concentrations (0, 25, 50, 75 and 100 mg mL^−1^) was carried out. Seeds of moso bamboo were soaked in sesame extracts to investigate their germination and growth. The allelochemicals were isolated and identified using the high performance liquid chromatograph (HPLC) system. The germination indices of the same organ decreased with the increase of extract concentrations, while the mean germination time increased, picking at the concentration of 100 mg mL^−1^. The radicle length and plumule length decreased, while the ratio of radicle length to plumule length increased. The allelopathy inhibition effect increased with the increase of extract concentrations, and it was significantly higher at the concentration of 100 mg mL^−1^ than that of 25 mg mL^−1^. The synthesis effect increased with the increase of extract concentrations, and it was significantly higher in leaf than root and stem. Chemical analyses identified 9 allelochemicals species (mostly phenolics and alkaloids) in the aqueous extracts. These results indicated that aqueous extracts of sesame caused the delay in seed germination and growth of moso bamboo, and phenolics and alkaloids in the aqueous extracts maybe the major reasons for the observed inhibition effects of sesame.

## Introduction

Moso bamboo (*Phyllostachys edulis* (Carriere) J. Houz.) is one of the most important forest resources in China, which has high economic, ecological and social values^1–4^. As a typical clonal plant, moso bamboo can rapidly and continuously expand to the neighbor system by its strong reproduction and spatial expansion ability of the rhizome-root system^5–7^. The annual expansion length of the rhizome can reach 2–6 m^8,9^. However, the rapid expansion of moso bamboo forest has posed a huge threat to the adjacent ecosystem and biological resources, leading to the loss of species diversity^10,11^, the destruction of native forest ecosystems^12,13^, the difficulty of natural regeneration^14,15^ and the stagnation of community succession^5,16^. In addition, the phenomenon of abandonment caused by the decrease of the bamboo shoot price and timber price and the increase of the labor costs reduced the enthusiasm of bamboo farmers for management^7,17^, which resulted in the expansion of moso bamboo to adjacent systems due to the fierce internal competition. Therefore, moso bamboo forest is considered to be invasive and has resulted in intense forest expansion^17,18^.

Previous study indicated that the expansion area of moso bamboo may be limited by physical barriers, which restricted the speed and range of rhizome growth^7^. Cai et al. put forward the method of “digging pit and irrigating water” to restrain the expansion of moso bamboo based on the growth characteristics of rhizome^19^. Suzuki and Nakagoshi proposed to increase the numbers of shoot harvesting and culm cutting to inhibit bamboo expansion^17^. Although these management strategies were effective for bamboo control, it was difficult to be applied in practice due to the disadvantages of long cycle, high investment and slow effectiveness. Therefore, new technical measures should be explored to control the expansion of moso bamboo forest.

Allelopathy is a direct or indirect effect caused by one plant (including microorganisms) on another through the production of chemical compounds that escape into the environment^20,21^. Allelochemicals are plant metabolites or their products that are released into the environment, which may affect plants at different stages of plant growth and development, such as seed germination, seedling growth and development, flowering and fruiting, vegetation formation and succession, species regeneration^22–25^. The huge number allelopathic interactions are typically negative in character, with positive relations being rare^26^. Allelochemicals affect germination and growth of neighboring plants by disruption of various physiological processes including photosynthesis, respiration, water and hormonal balance^26^. Sesame (*Sesamum indicum* L.) is an important oil crop, which has been widely used in traditional agricultural production in China to prevent weeds^27^. When sesame was planted around the bamboo forest, bamboo could not grow outside through sesame^27^. Therefore, the inhibition effect of sesame on bamboo expansion may exist, but few studies have been conducted on the related research.

Seed germination is the most important stage in plant growth and development^28,29^. Allelochemicals can be presented in every organ of plant parts such as roots, rhizomes, leaves, stems, bark, flowers, fruits and seeds^20,30,31^. The allelochemicals of sesame released to the environment through stem and leaf leachates, root exudates and residue decomposition liquid, may have allelopathic effect on seed germination and seedling growth of recipient plants^27^. Numerous studies were accomplished about the allelopathic effects of sesame on the germination and growth of other plants. Duary reported that sesame leaf extracts in different concentrations had allelopathic influence on germination, seedling growth and dry matter production of black gram (*Vigna mungo* L.) and rice (*Oryza sativa* L.)^32^. Soleymani and Shahrajabian found that sesame extracts caused the delay in the growth and germination of canola (*Brassica napus*)^33^.

Thus, we hypothesized that sesame extracts had allelopathic effects on moso bamboo according to the previous studies. In this study, we investigated the effects of sesame extracts on seed germination and growth of moso bamboo, and isolated and identified the potential allelochemicals, with the aims to prove the existence of allelopathic effects of sesame on bamboo growth and to provide a theoretical basis for restraining bamboo expansion.

## Results

### Germination index

The aqueous extracts of sesame significantly affected the germination indices of moso bamboo seeds (Table 1). The germination indices of the same organ decreased with the increase of the extract concentrations. The germination rate at 0 mg mL^−1^ was significantly higher than 25 mg mL^−1^ of stem and leaf extracts (*P* < 0.05), while no significant difference was found between 0 and 25 mg mL^−1^ of root extracts (*P* > 0.05). At the same organ, the germination vigor and germination index at 0 mg mL^−1^ were significantly higher than that of other concentrations, respectively (*P* < 0.05). However, no significant difference was found between 25 and 50 mg mL^−1^ at the same organ (*P* > 0.05). There was no significant difference between 0 and 25 mg mL^−1^ of root extracts (*P* > 0.05), while significant difference was found between 0 and 25 mg mL^−1^ of stem and leaf extracts, respectively (*P* < 0.05). At the same concentration, no significant difference was found among three organs except vigor index (*P* > 0.05). The vigor index of root extract at 100 mg mL^−1^ was significantly higher than the stem and leaf extracts (*P* < 0.05).

**Table 1:** Effects of different aqueous extracts of sesame on the germination indices.

Organ	Concentration (mg mL^−1^)	Germination rate (%)	Germination energy (%)	Germination index	Vigor index
Root	0	49.33 ± 4.62 Aa	42.67 ± 6.11 Aa	25.53 ± 2.00 Aa	5.14 ± 0.49 Aa
	25	46.67 ± 5.77 Aa	38.00 ± 2.00 Ba	20.95 ± 1.71 Ba	4.88 ± 0.55 Aa
	50	42.67 ± 1.15 Ba	38.00 ± 3.46 Ba	18.84 ± 1.82 Ba	3.57 ± 0.09 Ba
	75	37.33 ± 2.31 Ca	30.67 ± 4.16 Ba	15.82 ± 1.41 Ca	3.02 ± 0.61 Ba
	100	36.00 ± 5.29 Ca	29.33 ± 4.62 Ca	15.12 ± 2.64 Ca	2.95 ± 0.15 Ba
Stem	0	49.33 ± 4.62 Aa	42.67 ± 6.11 Aa	25.53 ± 2.00 Aa	5.14 ± 0.49 Aa
	25	44.67 ± 1.15 Ba	38.67 ± 1.15 Ba	22.29 ± 1.17 Ba	4.65 ± 0.27 Aa
	50	38.67 ± 3.06 Ba	37.33 ± 4.16 Ba	19.90 ± 1.62 Ba	3.62 ± 0.19 Ba
	75	38.00 ± 5.29 Ba	34.67 ± 4.62 Ba	17.23 ± 2.93 Ca	2.78 ± 0.38 Ca
	100	34.00 ± 4.00 Ca	32.00 ± 2.31 Ca	15.33 ± 2.28 Ca	2.67 ± 0.65 Cb
Leaf	0	49.33 ± 4.62 Aa	42.67 ± 6.11 Aa	25.53 ± 2.00 Aa	5.14 ± 0.49 Aa
	25	42.00 ± 3.46 Ba	36.00 ± 8.72 Ba	20.56 ± 1.47 Ba	4.02 ± 0.48 Ba
	50	41.33 ± 3.02 Ba	34.67 ± 3.06 Ba	18.83 ± 0.59 Ba	3.61 ± 0.65 Ba
	75	35.33 ± 2.47 Ba	30.00 ± 3.46 Ca	15.05 ± 1.66 Ca	2.91 ± 0.53 Ca
	100	30.00 ± 2.00 Ca	28.67 ± 5.03 Ca	12.99 ± 1.30 Ca	2.02 ± 0.12 Dc

Different capital letters of the same organ in the same column indicated significant differences among different concentrations at *P* < 0.05 level, and different lowercase letters of the same concentrations in the same column indicated significant differences among different organs at *P* < 0.05 level.

### Mean germination time

**Figure 1:**
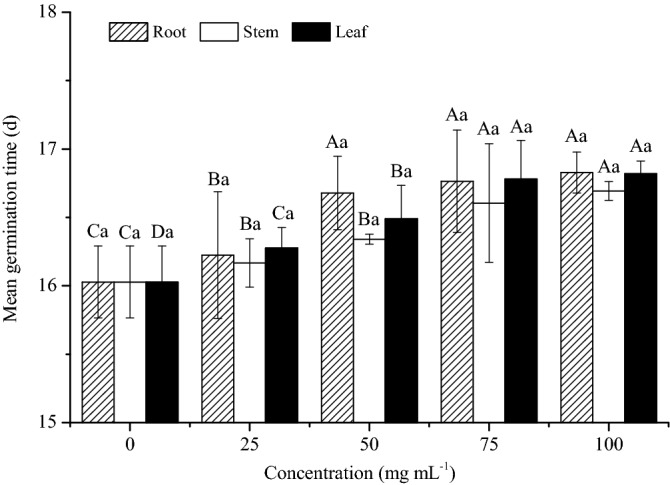
Effects of different aqueous extracts of sesame on the mean germination time. Different capital letters of the same organ indicated significant differences among different concentrations at *P* < 0.05 level, and different lowercase letters of the same concentrations indicated significant differences among different organs at *P* < 0.05 level.

The aqueous extracts of sesame significantly affected the mean germination time of moso bamboo seeds (Fig. 1). For the same organ, the mean germination time increased with the increase of the extract concentrations, and peaked at 100 mg mL^−1^. The mean germination time at 0 mg mL^−1^ was significantly lower than other treatments with aqueous extracts (*P* < 0.05). However, no significant difference was found between 75 and 100 mg mL^−1^ at the same organ (*P > *0.05). For the same concentration, the difference was not significant among root, stem and leaf (*P > *0.05). The mean germination time of root extract at 25 mg mL^−1^ was significantly lower than leaf extract (*P* < 0.05), while the mean germination times of root extract at 50, 75 and 100 mg mL^−1^ were significantly higher than leaf extract, respectively (*P* < 0.05).

### Growth characteristic

The aqueous extracts of sesame significantly affected the growth of moso bamboo (Table 2). For the same organ, the radicle length and plumule length decreased with the increase of the extract concentrations, respectively, while the ratio of radicle length to plumule length showed an opposite tendency. The radicle length and plumule length at 0 mg mL^−1^ were significantly higher than those at 50, 75 and 100 mg mL^−1^, respectively (*P* < 0.05). However, no significant difference was found between 0 and 25 mg mL^−1^ at the same organ (*P > *0.05). For root and leaf extracts, the ratio of radicle length to plumule length at 100 mg mL^−1^ was significantly higher than other concentrations (*P* < 0.05), and no significant difference was found among all concentrations for stem extracts (*P > *0.05).

**Table 2:** Effects of different aqueous extracts of sesame on seedling growth.

Organ	Concentration (mg mL^−1^)	Radicle length (cm)	Plumule length (cm)	Radicle/plumule
Root	0	5.95 ± 0.16 Aa	4.85 ± 0.09 Aa	1.22 ± 0.04 Ca
	25	5.80 ± 0.64 Aa	4.67 ± 0.36 Aa	1.24 ± 0.06 Ba
	50	5.40 ± 0.51 Ba	4.16 ± 0.12 Ba	1.30 ± 0.09 Ba
	75	4.89 ± 0.20 Ca	3.45 ± 0.30 Ca	1.42 ± 0.07 Ba
	100	4.08 ± 0.72 Da	2.84 ± 0.48 Da	1.44 ± 0.15 Aa
Stem	0	5.95 ± 0.16 Aa	4.85 ± 0.09 Aa	1.22 ± 0.04 Aa
	25	5.78 ± 0.49 Aa	4.65 ± 0.49 Aa	1.25 ± 0.03 Aa
	50	5.29 ± 0.27 Ba	4.09 ± 0.15 Ba	1.29 ± 0.08 Aa
	75	4.71 ± 0.33 Ca	3.46 ± 0.35 Ca	1.36 ± 0.10 Aa
	100	4.06 ± 0.74 Da	2.92 ± 0.50 Da	1.39 ± 0.09 Aa
Leaf	0	5.95 ± 0.16 Aa	4.85 ± 0.09 Aa	1.22 ± 0.04 Ca
	25	5.71 ± 0.31 Aa	4.52 ± 0.32 Aa	1.27 ± 0.03 Ba
	50	5.13 ± 0.69 Ba	3.96 ± 0.28 Ba	1.29 ± 0.18 Ba
	75	4.76 ± 0.34 Ca	3.51 ± 0.23 Ca	1.36 ± 0.02 Ba
	100	3.97 ± 0.14 Da	2.77 ± 0.53 Da	1.44 ± 0.43 Aa

Different capital letters of the same organ in the same column indicated significant differences among different concentrations at *P* < 0.05 level, and different lowercase letters of the same concentrations in the same column indicated significant differences among different organs at *P* < 0.05 level.

### Allelopathic index

The aqueous extracts of sesame significantly affected the allelopathic indices of moso bamboo (Table 3). The allelopathic indices were less than 0, illustrating that the extracts of sesame inhibited seed germination of moso bamboo. The allelopathy inhibition effect increased with the increase of the extract concentrations in the same organ, and it was significantly higher at the concentration of 100 mg mL^−1^ than that of 25 mg mL^−1^ (*P* < 0.05). For germination rate, no significant differences were found between 75 and 100 mg mL^−1^ root extracts, between 50 and 75 mg mL^−1^ stem extracts, and between 25 and 50 mg mL^−1^ leaf extracts (*P > *0.05). For radicle length and plumule length, significant differences of allelopathic index were found among different concentrations in the same organ (*P* < 0.05). For germination rate, the allelopathy inhibition effect of leaf extract was higher than that of root extract in the same concentration.

**Table 3:** Effects of different aqueous extracts of sesame on the allelopathic index.

Organ	Concentration (mg mL^−1^)	Germination rate (%)	Germination energy (%)	Germination index (%)	Vigor index (%)	Radicle length (%)	Plumule length (%)
Root	25	− 5.41 ± 0.58 Cc	− 10.94 ± 0.93 Bb	− 17.94 ± 0.47 Db	− 5.14 ± 0.58 Cb	− 2.42 ± 0.26 Db	− 3.85 ± 0.26 Db
	50	− 13.51 ± 1.14 Bc	− 10.94 ± 1.07 Bc	− 26.21 ± 0.69 Ca	− 30.65 ± 2.34 Ba	− 9.13 ± 0.40 Cc	− 14.18 ± 0.38 Cc
	75	− 24.32 ± 1.96 Ab	− 28.13 ± 2.25 Aa	− 38.01 ± 1.02 Bb	− 41.30 ± 3.03 Aa	− 17.80 ± 0.78 Bb	− 28.81 ± 0.76 Ba
	100	− 27.03 ± 2.83 Ac	− 31.25 ± 2.86 Aa	− 40.78 ± 1.10 Ab	− 42.55 ± 2.54 Ac	− 25.99 ± 1.18 Ab	− 38.79 ± 1.16 Aa
Stem	25	− 9.46 ± 1.03 Cb	− 9.38 ± 0.86 Db	− 12.68 ± 0.35 Dc	− 9.50 ± 0.92 Cb	− 2.72 ± 0.26 Db	− 4.20 ± 0.26 Db
	50	− 21.62 ± 1.18 Ba	− 12.50 ± 1.03 Cb	− 25.22 ± 0.70 Ca	− 29.59 ± 1.69 Ba	− 11.04 ± 0.48 Cb	− 15.77 ± 0.41 Cb
	75	− 22.97 ± 2.06 Bb	− 18.75 ± 1.39 Bb	− 32.50 ± 0.86 Bc	− 45.88 ± 3.24 Aa	− 20.81 ± 0.93 Ba	− 28.60 ± 0.75 Ba
	100	− 31.08 ± 2.86 Ab	− 25.00 ± 2.71 Ab	− 39.96 ± 1.08 Ab	− 48.15 ± 3.71 Ab	− 31.63 ± 1.46 Aa	− 40.69 ± 1.10 Aa
Leaf	25	− 14.86 ± 1.25 Ca	− 15.63 ± 1.39 Ba	− 19.47 ± 0.51 Da	− 21.79 ± 1.36 Ca	− 3.90 ± 0.26 Da	− 6.88 ± 0.27 Da
	50	− 16.22 ± 2.06 Cb	− 18.75 ± 1.46 Ba	− 26.22 ± 0.69 Ca	− 29.85 ± 1.28 Ca	− 13.69 ± 0.59 Ca	− 18.33 ± 0.48 Ca
	75	− 28.38 ± 2.59 Ba	− 29.69 ± 2.64 Aa	− 41.04 ± 1.11 Ba	− 43.32 ± 2.75 Ba	− 19.87 ± 0.88 Ba	− 27.73 ± 0.73 Ba
	100	− 39.19 ± 2.93 Aa	− 32.81 ± 3.72 Aa	− 49.09 ± 1.35 Aa	− 60.76 ± 3.83 Aa	− 33.28 ± 1.54 Aa	− 42.59 ± 1.16 Aa

Different capital letters of the same organ in the same column indicated significant differences among different concentrations at *P* < 0.05 level, and different lowercase letters of the same concentrations in the same column indicated significant differences among different organs at *P* < 0.05 level.

### Synthesis effect

**Figure 2:**
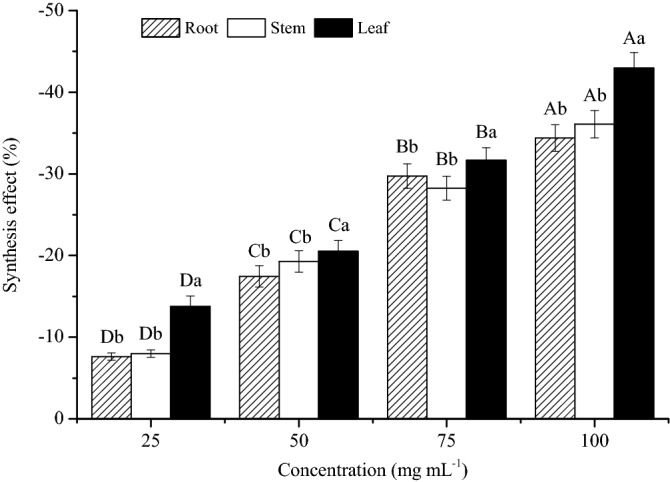
Effects of different aqueous extracts of sesame on the synthesis effect. Different capital letters of the same organ indicated significant differences among different concentrations at *P* < 0.05 level, and different lowercase letters of the same concentrations indicated significant differences among different organs at *P* < 0.05 level.

The synthesis effect was significantly affected by the aqueous extracts of sesame (Fig. 2). For the same organ, the synthesis effect increased with the increase of the extract concentrations, and peaked at 100 mg mL^−1^. The synthesis effect at 25 mg mL^−1^ was significantly lower than other extract concentrations (*P* < 0.05). For the same concentration, the synthesis effect of leaf was significantly higher than that of root and stem (*P* < 0.05), while no significant difference was found between root and stem (*P > *0.05).

### Chemical profiling of allelochemicals

The relative contents of allelochemicals in the aqueous extracts of sesame were showed in Table 4. Nine allelochemicals species were identified in the initial HPLC analysis, most of which were found at significantly lower contents. The relative contents of phenolics and alkaloids were significantly higher than other species (*P* < 0.05), and no significant difference was found among the three organs (*P > *0.05).

**Table 4:** Relative content of allelochemicals in the aqueous extracts of sesame.

Allelochemical species	Root (%)	Stem (%)	Leaf (%)
Terpenoids	9.37 ± 0.09 Cb	12.13 ± 0.50 Ca	8.63 ± 1.06 Cb
Flavonoids	4.60 ± 1.11 Db	8.84 ± 3.29 Ca	4.09 ± 0.28 Db
Phenolics	45.34 ± 5.97 Aa	48.68 ± 2.94 Aa	45.52 ± 9.12 Aa
Cinnamic acid and its derivatives	0.03 ± 0.01 Fa	0.04 ± 0.01 Da	0.04 ± 0.01 Ea
Quinines	0.53 ± 0.24 Ec	0.70 ± 0.09 Db	1.05 ± 0.36 Ea
Steroid and its derivatives	3.06 ± 0.45 Eb	2.97 ± 0.12 Db	8.85 ± 1.02 Ca
Hydroxyl and substituted benzoic acids	0.06 ± 0.01 Eb	0.04 ± 0.00 Db	0.12 ± 0.03 Ea
Alkaloids	34.22 ± 4.79 Ba	24.45 ± 5.52 Ba	30.10 ± 7.37 Ba
Coumarins	2.78 ± 0.59 Ea	2.15 ± 0.78 Da	1.60 ± 0.40 Ea

Different capital letters of in the same column indicated significant differences among different allelochemical species at *P* < 0.05 level, and different lowercase letters of in the same row indicated significant differences among different organs at *P* < 0.05 level.

## Discussion

Seed germination is the basis of plant growth, which directly affects the survival and development of plants^28,34,35^. Seed germination test is one of the most commonly used biological detection methods in allelopathy research^20^. In the natural state, water is the natural solvent of plants, which can leach out the chemicals in plants^36^. Numerous studies have documented that the allelochemicals released by sesame can lead to a significant reduction in the seed germination and seedling growth^32,37^. In our study, the allelopathic effects of sesame extracts on seed germination and seedling growth of moso bamboo were studied.

In the present research, the germination indices of moso bamboo seeds changed obviously after soaking with sesame extracts. The germination rate decreased with the increase of the aqueous extract concentrations, which was consistent with the results of a previous study by Amare, who found that the germination rate was directly related to extract concentrations^38^. However, many studies have found an interesting phenomenon, namely promotional effect at low concentration and inhibitory effect at high concentration^39–41^. In our study, the allelopathy of sesame extracts on the germination of moso bamboo seeds did not show promoting effect at low concentration, which may be related to the high concentration gradient. In addition, the mean germination time increased with the increase of aqueous extract concentrations, illustrating that the aqueous extracts of sesame delayed the germination process of moso bamboo seeds. The same pattern was reported by Alencar and Lozano-Isla et al., who found a strong and negative correlation between germination rate and mean germination time^42,43^. A possible explanation for this phenomenon was presented by Yan et al., who found that the allelochemicals affected the metabolism of substances and the activity of various enzymes in the process of seed germination, which resulted in seed deterioration and the decrease of vigor^40^.

The growth and development of radicle directly affect the growth of plants, while the growth of plumule directly reflects the growth rate of plants at seedling stage. Researches showed that root length was more sensitive to allelopathy than seedling height in seed germination^22,44^. In this study, the lengths of radicle and plumule showed a decline trend with the increase of aqueous extract concentrations. It was consistent with the results of a previous study by Sahu and Devkota, who observed that the aqueous extract of leaves from *Mikania micrantha* significantly inhibited the root and shoot growth of *Oryza sativa*^45^. However, our result was inconsistent with some previous reports, which showed a promotion effect at low concentration on the growth of radicle and plumule^25,46^. The differences in the results may be attributed to the high concentration gradient in this study. Although the lengths of radicle and plumule decreased with the increase of aqueous extract concentrations, the ratio of radicle length to plumule length increased. The increasing ratio of radicle length to plumule length is a protective effect of plant under the adversity stress, which is conducive to promoting its absorption of water and nutrients and alleviating the damage caused by the adversity stress^47^.

In our study, the allelopathic indices were less than 0, illustrating that the aqueous extracts of sesame inhibited seed germination and growth of moso bamboo. The allelopathy inhibition effect increased with the increase of the extract concentrations in the same organ. This result was consistent with Jiang et al., who found the same trend in two herb species as affected by root exudates from *Picea asperata*^48^. In addition, the synthesis effects were also less than 0, and their absolute values increased with the increase of extract concentrations. The same result was also observed by Huang et al., who found a negative synthesis effect of *Cinnamomum septentrionale* leaf litter on *Eucalyptus grandis* saplings^21^.

Many studies showed that allelochemicals could be classified into numerous categories according to their different structures and properties^20,26,30^. In our study, 9 allelochemicals species were identified in the aqueous extracts, and the relative contents of phenolics and alkaloids were the highest. Phenolics are among the most common classes of compounds exuded^49^, and our findings indicate that sesame is no exception. Most phenolics can stimulate indoleacetic acid (IAA) oxidase activity and inhibit the reaction of peroxidase (POD) with IAA, bound gibberellin (GA) or IAA to influence endogenous hormone levels^50^. Previous study showed that the decrease of germination ability was significantly related to the increase of membrane damage^51^. In this study, phenolic compounds were the highest, which increased the lipid peroxidation of cell membrane and damaged the cell membrane^52^. Alkaloids are also known for their allelopathic effect, which have been reported to affect DNA synthesis, respiration, and electron transport^49^. In summary, phenolics and alkaloids maybe the major reasons for the observed inhibiting effects of sesame.

## Conclusion

This study clearly demonstrated that the allelopathic effects of sesame extracts on seed germination and growthof moso bamboo. With the increase of extract concentrations, the germination indices and growth charactetistics decreased, while the allelopathy inhibition effect and the synthesis effect increased. These results indicated that the aqueous extracts of sesame significantly inhibited seed germination and growth of moso bamboo, which provided theoretical basis for inhibiting its expansion. Numerous allelochemicals were found in the aqueous extracts, and phenolics and alkaloids should be the potential allelochemicals of sesame inhibiting the expansion of moso bamboo forest. However, the expansion of moso bamboo was mainly conducted the rhizome-root system. Therefore, further research should be conducted to explore the allelopathic effects of sesame extracts on rhizome-root system of moso bamboo, and to provide effective measures to control the expansion of moso bamboo.

## Materials and methods

### Seed and plant materials

Seeds of moso bamboo were collected from Guilin City, Guangxi Province in September 2019 and stored at room temperature. The thousand-seed weight was 26.57 g. The plants of sesame were collected from the Bamboo Botanical Garden, Zhejiang Academy of Forestry in October 2019. The plants must be well growth, and the whole plant should be collected.

### Preparation of aqueous extracts

The plants of sesame were separated into roots, stems and leaves. After washing away the soil and impurities, the roots and stems were cut into small segments of 1 cm, and the leaves were divided into small segments of 2 cm^2^. Then, 100 g fresh samples (root, stem and leaf) were kept in a conical flask with 1 L distilled water separately. The conical flask was shaken regularly (every 6 h) and left for 24 h at room temperature. The mixtures were then filtered through muslin cloth and filter paper, and the aqueous extracts of roots, stems and leaves with the concentration of 100 mg mL^−1^ were obtained. Aqueous extracts of 75, 50 and 25 mg mL^−1^ concentrations were obtained by diluting the original extract (100 mg mL^−1^) with distilled water. The manufactured solutions of aqueous extracts were placed in the refrigerator at 4 °C for seed germination.

### Seed germination

The germination experiment of moso bamboo seeds was conducted in Zhejiang Provincial Key Laboratory of Bamboo Research according to the inspection regulations of International Seed Testing Association (ISTA) in October 2019. The experiment was a factorial design with three organs (root, stem and leaf) and five concentrations (0, 25, 50, 75 and 100 mg mL^−1^).

Prior to the experiment, the bamboo seeds without any pest infestation were carefully selected and soaked in distilled water for 24 h before being sterilized with the KMnO_4_ solution (0.3%) for 15 min. The seeds were then rinsed with sterile distilled water for 3 times and air-dried in a clean bench. The Petri dish (15 cm in diameter) and filter paper were also sterilized at high temperature (105 °C) for 20 min before use. Two pieces of filter paper were placed into the Petri dish containing 10 mL of distilled water or different concentrations of the prepared aqueous extracts. Then, 50 seeds were laid uniformly in each Petri dish. Each treatment was repeated thrice. All the Petri dishes were placed in an illuminated incubator with a constant temperature of 25 °C and a constant humidity of 90%. Water consumption in the Petri dish was measured by weighing method, and the extra water was supplemented to original weight. The germination bed was changed every 4 days. Dates on seeds germination each day were recorded, and the lengths of radicle and plumule were measured with a digital caliper. When the number of germinated seeds was less than 1% of the total number of tested seeds for 3 consecutive days, the germination experiment finished.

### Calculation methods

The germination rate (GR), germination energy (GE), germination index (GI), vigor index (VI) and mean germination time (MGT) were calculated by the following equation^47,48^.

1 $$GR \left(\%\right)=\frac{Ni}{N}\times 100$$

2 $$GE \left(\mathrm{\%}\right)=\frac{Nt}{N}\times 100$$

3 $$GI=\sum (d/n)$$

4 $$VI=GR\times (LR+LP)$$

5 $$MGT=\frac{\sum (d\times n)}{\sum n}$$

where *Ni* is the number of germinated seeds in the 28th day, *N* is the total seed number in the petri dish, *Nt* is the number of germinated seeds when the daily germination number reaches the peak, *d* is the number of seeds emerging on a given day, *n* is the time after setting the seeds for germination, *LR* is the average length of radicle in the 28th day, and *LP* is the average length of plumule in the 28th day.

The allelopathy index (RI) was calculated by the following equation^53^:

6 $$RI (\mathrm{\%})=\frac{(T-C)}{C}\times 100$$

where *T* is the treatment value and *C* is the control value.

The synthesis effect (SE) was represented by the average of the RI of GR, GE, GI, VI, LR and LP under the same treatment^48^.

The ratio of radicle length to plumule length (Radicle/Plumule) was calculated to reflect the balance between the two parts of seedlings.

### Isolation and identification of allelochemicals

After observing evidence of allelopathy in the aqueous extract of sesame, steps were taken to identify active compounds. The aqueous extracts (100 mg mL^−1^) were freeze-dried, and 20 mg aliquot of the freeze-dried samples were precise weighed and were transferred to an Eppendorf tube, after addition of 500 μL of extract solution (methanol/water = 3:1, precooled at – 40 °C, containing internal standard). After 30 s vortex, the samples were homogenized at 35 Hz for 4 min and sonicated for 5 min in ice-water bath. Repeat homogenize and sonicate for 3 times. The samples were extracted over night at 4 °C on a shaker,and then centrifuged at 12,000 rpm for 15 min at 4 °C. The supernatant was filtered through a 0.22 μm microporous membrane, then the resulting supernatants were diluted 10 times with methanol/water mixture (v:v = 3:1, containing internal standard) and vortexed for 30 s. The allelochemicals were isolated and identified using the high performance liquid chromatograph (HPLC) system (ExionLC AD, Sciex, USA).

### Statistical analysis

One-way analysis of variance (ANOVA) and least significant difference test (LSD) were used to examine the effects of sesame extracts on seed germination indices, growth characteristics, allelopathy index and relative contents of allelochemicals. The statistical significance was evaluated at *P* < 0.05 level. All statistical analyses were conducted in the SAS 9.0 software.

### Ethics approval

The collection of bamboo seeds was permitted by local famers orally and the collection of sesame was permitted by the staff of Bamboo Botanical Garden. The study complied with local (Zhejiang Province) and national (China) regulations. All the methods in this manuscript were carried out in accordance with relevant guidelines and regulations.
